# Effects of moderate-intensity intermittent hypoxic training on health outcomes of patients recovered from COVID-19: the AEROBICOVID study protocol for a randomized controlled trial

**DOI:** 10.1186/s13063-021-05414-2

**Published:** 2021-08-12

**Authors:** Átila Alexandre Trapé, Marta Camacho-Cardenosa, Alba Camacho-Cardenosa, Eugenio Merellano-Navarro, Jhennyfer Aline Lima Rodrigues, Elisangela Aparecida da Silva Lizzi, Carlos Arterio Sorgi, Marcelo Papoti, Javier Brazo-Sayavera

**Affiliations:** 1 School of Physical Education and Sport of Ribeirão Preto, University of Sao Paulo (USP), Ribeirão Preto, SP Brazil; 2 Ribeirão Preto College of Nursing, USP, Ribeirão Preto, SP Brazil; 3 Faculty of Languages and Education, University of Nebrija, Madrid, Spain; 4 Faculty of Sport Science, University of Extremadura, Cáceres, Spain; 5 Grupo de Investigación EFISAL, Universidad Autónoma de Chile, Talca, Chile; 6 Ribeirão Preto Medical School, USP, Ribeirão Preto, SP Brazil; 7 Academic Department of Mathematics, Federal University of Technology, Paraná, Cornélio Procópio, PR Brazil; 8 Faculty of Philosophy, Sciences and Letters of Ribeirão Preto, USP, Ribeirão Preto, SP Brazil; 9 School of Physical Education and Sport of Ribeirão Preto, University of Sao Paulo (USP), Ribeirão Preto, SP Brazil; 10 Department of Sports and Computer Science, Universidad Pablo de Olavide (UPO), Seville, Spain; 11 PDU EFISAL, Centro Universitario Regional Noreste, Universidad de la República, Rivera, Uruguay

**Keywords:** Exercise, Hypoxia-inducible factor 1 alpha subunit, Inflammation, Respiratory function tests, SARS virus

## Abstract

**Background:**

Recent studies point to a lower number and reduced severity of cases in higher altitude cities with decreased oxygen concentration. Specific literature has shown several benefits of physical training, so, in this sense, physical training with hypoxic stimulus appears as an alternative that supports the conventional treatments of the COVID-19 patient’s recovery. Thus, this study’s primary aim is to analyze the effects of moderate-intensity intermittent hypoxic training on health outcomes in COVID-19 recovered patients.

**Methods:**

A clinical trial controlled double-blind study was designed. Participants (30–69 years old) will be recruited among those with moderate to severe COVID-19 symptoms, approximately 30 days after recovery. They will be included in groups according to the training (T) and recovery (R) association with hypoxia (H) or normoxia (N): (a) T_H_:R_H_, (b) T_N_:R_H_, (c) T_N_:R_N_, and last (d) the control group. The 8-week exercise bike intervention will be carried out with a gradual load increase according to the established periods, three times a week in sets of 5 min, 90 to 100% of the anaerobic threshold (AT), and a 2.5-min break. Blood will be collected for genotyping. First, after 4 weeks (partial), after 8 weeks, and later, 4 weeks after the end of the physical training intervention, participants will perform assessments. The primary outcome is the maximum oxygen consumption (VO_2_peak). The secondary outcomes include lung function, inflammatory mediators, hematological, autonomic parameters, AT, body composition analysis, quality of life, mental health, anthropometric measurements, and physical fitness. The statistical analysis will be executed using the linear regression model with mixed effects at a 5% significance level.

**Discussion:**

This study is designed to provide evidence to support the clinical benefits of moderate-intensity intermittent hypoxic training as a part of the treatment of patients recovered from COVID-19. It may also provide evidence on the efficacy and safety of intermittent hypoxic training in different health conditions. Lastly, this study presents an innovative strategy enabling up to 16 participants in the same training session.

**Trial registration:**

ClinicalTrials.gov RBR-5d7hkv. Registered after the start of inclusion on 3 November 2020 with the Brazilian Clinical Trials Registry

**Supplementary Information:**

The online version contains supplementary material available at 10.1186/s13063-021-05414-2.

## Background

In March 2020, the World Health Organization raised the state of COVID-19 contamination to the pandemic. Currently, infections have exceeded 175 million cases and 3.8 million deaths. News related to COVID-19 is commented on daily in the media and has caused great concern and impact on Global Public Health [1].

Symptoms presented by those infected with COVID-19 are common to other respiratory infections. They may include fever, cough, sore throat, headache, fatigue, muscle pain, loss of smell, and shortness of breath. The clinical condition is diverse, ranging from an asymptomatic state to acute respiratory syndrome and damage to various body systems, increasing inflammatory markers, cardiovascular alterations, and injuries to the lungs and kidneys. The worldwide mortality rate of COVID-19 ranges from 2 to 3%, so most people recover from the disease. However, it is important to highlight that a significant part of this recovered population presents persistent symptoms or sequelae after the recovery; thus, they could be investigated [2–4].

Recent studies indicate that people who live at altitude with low levels of oxygen (O_2_) have a lower prevalence of COVID-19 and less severity in cases of infection [5–10]. The factors that may be related to lower susceptibility to COVID-19 involve physiological and anatomical adaptations in the lungs, with perfusion and capacity improvements, but mainly hypoxia-inducible-factor-1α (HIF-1α) activation [5–7], which has become scientifically relevant due to its discovery behind the Nobel Prize in Physiology or Medicine in 2019 [11]. Furthermore, according to the phenomenon of hormesis, moderate doses of intermittent hypoxia would lead to the activation of HIF-1α, triggering positive adaptations in the face of different diseases [12].

Specifically concerning COVID-19, HIF-1α can decrease the expression of the angiotensin-converting enzyme (ACE)2, which is indicated as a facilitator of the SARS-CoV2 virus into cells, mainly in the lungs [6, 7, 13]. HIF-1α production increases the expression of ACE1, stimulating the formation of angiotensin II, which regulates angiotensin II type 1 receptor (AGTR1). This process would reduce the expression of ACE2 [5, 6]. The infection with SARS-CoV2 leads to the destruction of epithelial cells, representing an important part of airway immunity. Consequently, a series of changes in immunological and inflammatory markers is triggered [14, 15]. Still, it is important to note that HIF-1α can increase the gene expression to produce erythropoietin (EPO), countering the harmful effects of covid-19. Among EPO’s benefits are highlighted: neuroprotection, central ventilation stimulation, endothelium protection, and pulmonary vasodilation, red blood cell production, and anti-inflammatory effect [16].

On the other hand, moderate-intensity interval exercise can reduce chronic inflammation and strengthen the immune system [17–20], reducing the severity and mortality of viral diseases [21]. In addition, higher aerobic capacity can produce short-term improvements in the immune and respiratory systems [22], both affected by COVID-19 [23].

Training methods using hypoxia as a resource have existed since the 1960s to increase performance at sea level or generate acclimatization for athletes to improve their performance in altitude tests [24, 25]. More recent studies [25–30] have shown that this type of intervention can provide good health outcomes and that physical training under normobaric hypoxia is safe and can be performed with different populations. For example, studies developed with overweight and obese women showed that a reduction of fat mass has been reported with a concomitant increase in lean mass [26] and cardiorespiratory fitness [27]. These results were better in the groups that performed interval training or sprints in hypoxia than those trained in normoxia after 12 weeks (36 sessions). Also, it has been reported the effects on bone mineral density in older adults, but only the group that underwent training of whole-body vibration in hypoxia showed improvements after 18 weeks (36 sessions), while the group that trained in normoxia did not improve [28]. Additionally, in another study performed over 3 weeks (9 sessions), the aerobic capacity, HIF-1α, nitric oxide, and pro-angiogenic factors improved, but only in the group of active young men who performed high-intensity interval training under hypoxic conditions on a bicycle [29]. Moreover, a systematic review and meta-analysis identified that hypoxic training promoted better responses in reducing triglycerides and increasing muscle mass than the same exercise performed in normoxia [30].

Although the studies previously mentioned [26–29] had shown health benefits for participants right after the intervention, a recent study [31] that performed high-intensity training under hypoxic conditions found effects after 4 weeks of the end of the intervention. At that moment, the trunk fat mass of overweight and obese women decreased significantly compared to the intervention’s result. This result may indicate that the adaptations promoted by the hypoxic training can last longer since this improvement after 4 weeks from the end was not found in the group that trained in normoxia.

Even though exposure to physical training and hypoxia can result in several health benefits, the response magnitude can vary considerably between individuals. Genetic characteristics could explain this variation and the different reactions that patients recovered from COVID-19 present in the gradual recovery process. The COVID-19 number and severity of cases in some populations have been associated with ACE gene and the frequency of I allele (insertion) or D allele (deletion) of 287-bp, drawing attention to the activity of this protein, which has a relationship with these genetic variants [32]. However, the results are still preliminary and point to this relationship’s complexity, requiring further exploration. Furthermore, since the modulation of ACE2 by HIF-1α involves the regulation of AGTR1, the investigation of genetic variants related to this receptor may also be necessary to understand this context better [9, 10]. Genetic variation could also influence the response to training in hypoxia, and these analyses may explain why some people present more physical training benefits than others. For example, the DD genotype of ACE may be related to a worse adaptation in altitude, and the presence of the I allele may indicate a better possibility of adaptation [33]. There is also evidence about an association between EPO’s production and genetic variants of the promoter region of this gene [34].

Thus, we hypothesize that training in normoxia would promote improvements in health status comparing to the control group that will not train. Both groups that will train in hypoxia would present even better results than the normoxia group, helping in the faster and more complete recovery of the organism, restoring immune system homeostasis and cardiorespiratory capacity. Still, it is expected that the studied genetic variants would affect the magnitude of response to the intervention.

## Methods

This protocol was written in accordance with the Standard Protocol Items: Recommendations for Interventional Trials guidelines (SPIRIT) [35]. We used the SPIRIT checklist when writing our report [36].

### Aims

#### Primary aim

To analyze the effects of moderate-intensity intermittent hypoxic training on the health outcomes of patients recovered from COVID-19.

#### Secondary aims

To describe the health status post-COVID, patients recovered who had presented moderate to severe symptoms will be assessed through lung function (spirometry), hematological and serum biochemical (complete blood cell count, including hemoglobin, red blood cell, white blood cell, lymphocyte, neutrophil, and platelet; glucose; triglycerides, total cholesterol, and fractions; lactate dehydrogenase; liver enzymes, such as alanine aminotransferase and aspartate aminotransferase; and EPO), immunological (cytokines, such as IL-6, IL-8, IL-10, and TNF-α; and, lipid mediators, such as eicosanoids), and autonomic parameters (heart rate variability), in addition to physical fitness (motor tests), quality of life (SF – 12), and mental health (DASS – 21).

To analyze the effects of moderate-intensity intermittent hypoxic training in patients recovered from COVID-19 who presented moderate to severe symptoms on:

- Hematological parameters, using the complete blood cell count and other biochemical analyses; immunological ones through inflammation markers (cytokines and lipid mediators); and erythropoiesis through EPO analysis
- Physical fitness through aerobic power, aerobic capacity, strength resistance of lower limbs, agility, and dynamic balance
- Anthropometric measurements and body composition through the distribution of lean, bone, and adipose tissue
- Blood pressure and autonomic parameters through heart rate variability (HRV)
- Quality of life through 12-item Short-Form Health Survey (SF-12), and the state of mental health through the assessment of levels of depression, anxiety, and stress, using the Depression, Anxiety and Stress Scale (DASS - 21)

To determine the effects after 4 weeks of the end of the moderate-intensity intermittent hypoxic training on lung function, hematological, immunological, and autonomic parameters and physical fitness, quality of life, and mental health in COVID-19 recovered patients with previous moderate to severe symptoms.

To analyze the influence of genetic variants of ACE, AGTR1, a promoter region of the EPO gene, and the EPO receptor on the effects of moderate-intensity intermittent hypoxic training in COVID-19 recovered patients.

### Design and setting

The study design is based on a randomized, double-blind controlled clinical trial composed of four groups. The control group being formed by the participants who are not available to participate in the intervention and accept to carry out a follow-up through the evaluations; and the physical training groups randomly divided according to the association of training (T) and recovery (R) with hypoxia (H) or normoxia (N): (a) T_H_:R_H_, (b) T_N_:R_H_, and (c) T_N_:R_N_. The allocation ratio will be 1:1:1:1.

**Fig. 1:**
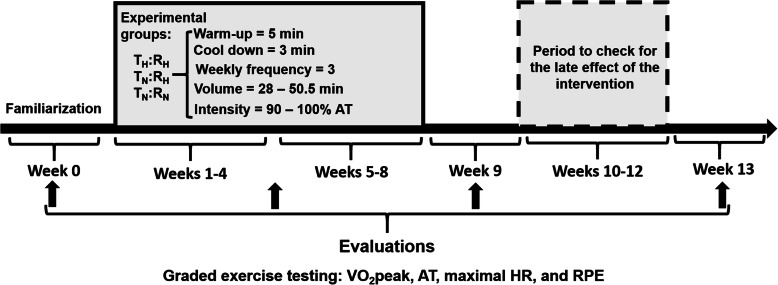
Experimental design. VO2peak, – maximal oxygen uptake; AT, - anaerobic threshold; HR, - heart rate; RPE, - the rate of perceived exertion

As Fig. 1 shows, the experimental protocol will consist of (i) familiarization and carrying out the initial assessments (evaluation 1) in the three sessions of week 0; (ii) 8-week intervention with a partial assessment to adjust the training load on week 5 (half of the intervention – evaluation, (2) (iii) reassessments on week 9, following the end of physical training intervention (evaluation 3), and (iv) reassessments on week 13, four after the end of the intervention (evaluation 4).

### Characteristics of participants

The inclusion criteria will be (1) participants aged between 30 and 69 years old and convalescent from COVID-19 (having the test with a positive diagnosis), (2) having moderate to severe symptoms, and (3) approximately 30 days since recovery from clinical signs or medical discharge (if they had been hospitalized). The exclusion criteria will be (1) exposure to high-altitude places > 1500 m in the last 3 months, (2) significant physical limitations to carry out assessments or intervention, (3) acute or chronic clinical illnesses without medical supervision, (4) anemia, (5) use of immunosuppressive drugs, (6) pregnant women, (7) hormone replacement, (8) smokers, (9) excessive use of alcohol or drugs, (10) three absents in sequence during the intervention, and (11) taking part in less than 75% of the total sessions planned.

### Instrumentation normobaric hypoxia

The three training groups, except the control, will carry out the training program in the same way: on bicycles, around the normobaric hypoxia tents (Colorado Altitude Tents, Colorado, USA) using the individual system that includes the face mask. Thus, participants will not be aware of the O_2_ concentration in the air contained in the tent in which they are positioned. This procedure will be done to minimize possible psychological influences from exposure to hypoxia.

**Fig. 2:**
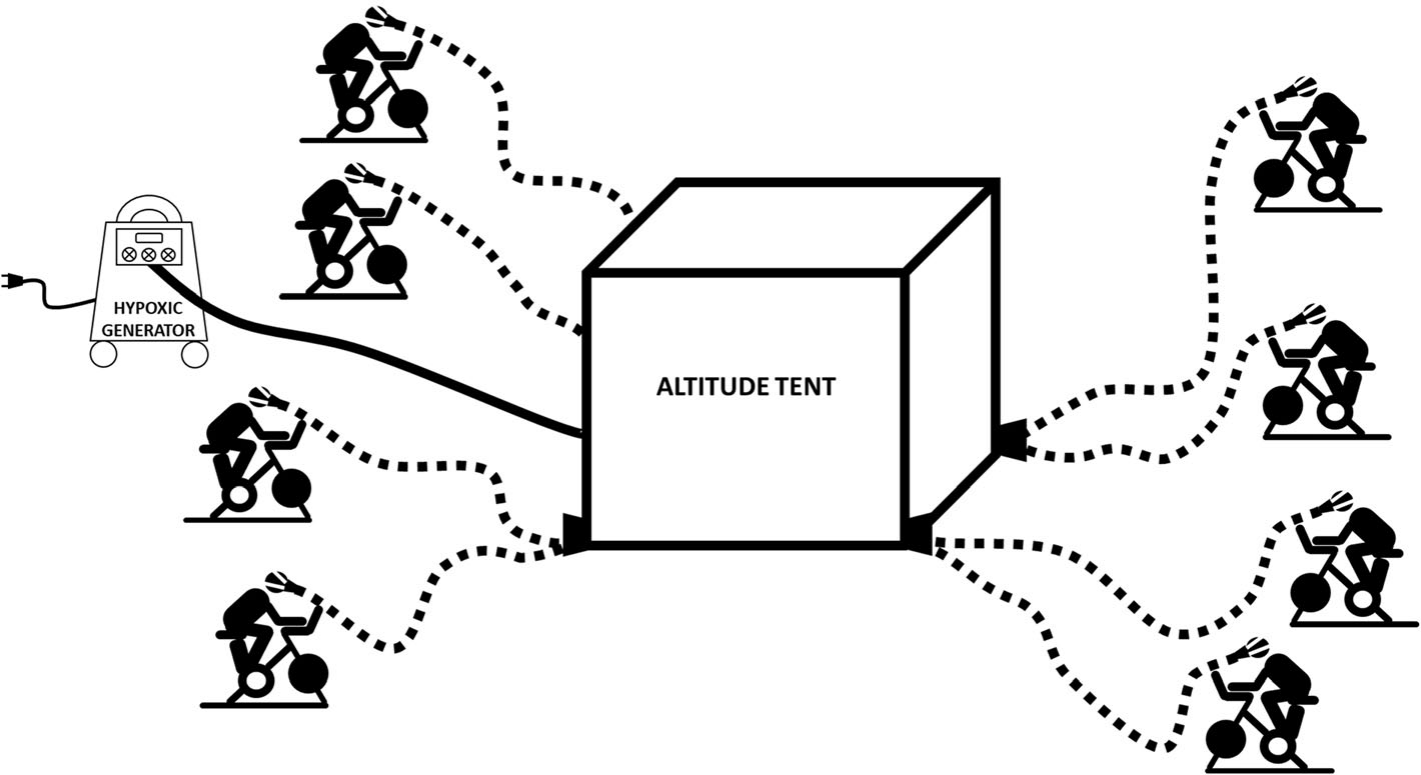
Strategy for training participants

A system that includes an individual face mask to be worn in the training program has been developed. Each participant will receive a mask for exclusive use in the initial evaluations and be instructed to wear it during the entire intervention aiming to work at the highest level of safety. Fig. 2 illustrates the strategy that will be used for the intervention. The normobaric hypoxia tents with O_2_ concentration control will be set up at the Multisport Gymnasium at the University. This space has good air circulation, and the bikes will be positioned around the tents, respecting 3 m between each bike. Up to eight participants will breathe the air in the normobaric hypoxia tents through hoses and an individual system that includes the mask. Two tents will be used simultaneously, enabling the training of up to 16 participants per period.

**Fig. 3:**
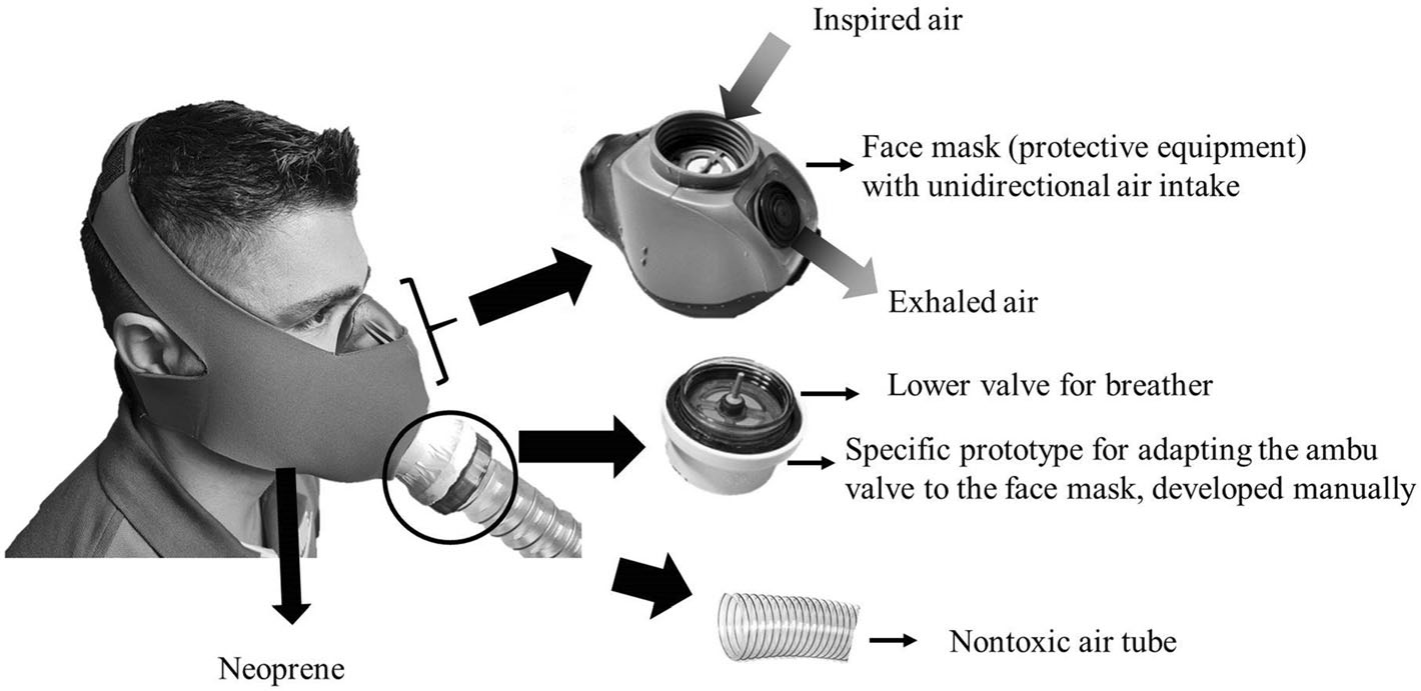
Individual mask for training participants

Each system will consist of a face mask of personal protective equipment with membranes that will allow only the inspired air to enter the upper cavity (nose) and only the exhaled air to exit the lower cavity (mouth). This membrane system will prevent air from being exhaled through the upper cavity, thus avoiding the possibility of contamination of the air contained in the tent, or else, inspired by the lower cavity, avoiding that the ambient air is inspired in this case, which would decrease the precision of the study. A safety valve will be used on the bottom of resuscitation bags to increase this strategy’s efficiency and facilitate the hose connection that will take the air from the normobaric hypoxia tent to the individual system (mask). The system will be covered with neoprene protection to provide greater comfort and stability during physical training (Fig. 3).

### Intervention protocol

The moderate training sessions in bicycles will be performed with a frequency of 3 times a week and consist of three parts (warm-up, main part, and cool down) with a total duration of up to 50.5 min. The initial stage (warm-up) and the final stage (cool down) will last for 5 and 3 min, respectively, and will be of low intensity, corresponding to “easy” by the rate of perceived exertion (RPE). The training intensity in the main part will be based on the AT values. Blood lactate concentration [La-], target heart rate, and RPE will control the load. The training will consist of sets with efforts lasting 5 min with an intensity close to the upper limit of training zone 2 (90–100% of AT) with a passive pause of 2.5 min between efforts. Training loads will be increased during the first 4 weeks, as it is explained in Table 1.

**Table 1:** Training dynamics week by week, the progression of the load, the total volume of each session, and internal training load (Trimp)

Training dynamics	W1	W2	W3	W4-W8
Number of sets	3	4	5	6
Efforts (min)	5	5	5	5
Time recovery between efforts (min)	2.5	2.5	2.5	2.5
Total volume of each session (min)	28	35.5	43	50.5
RPE	5	5	5	5
Trimp (AU)	140	177.5	215	252.5

*W* week, *RPE* the rate of perceived exertion, *AU* arbitrary units

Additionally, the internal training load (Trimp) will be quantified at each session. It will be assumed as the product between the duration of the session (minutes) by the RPE values, the results being expressed in arbitrary units (AU). The RPE will be presented after the end of each training session [37]. This procedure will monitor possible differences in the absolute internal load among the T_H_:R_H_, T_N_:R_H_, T_N_:R_N_ groups. An estimative is presented in Table 1 with a fixed RPE value of 5.

It is important to highlight that despite the intensity of the sessions being considered “moderate” and, theoretically allowing participants to carry out the proposed training continuously, we believe that the inclusion of 2.5 min between efforts, that is, an effort: pause relationship of 2:1 will allow less discomfort during efforts, without, however, compromising the aerobic “gains” from this training model. Other necessary adaptations will be made according to the performance and needs of each participant. The final part (cool down) will last for 3 min with “easy” intensity by the RPE.

Out of the three training groups, two will be exposed to hypoxia according to the association of training (T) and recovery (R) with hypoxia (H): (a) T_H_:R_H_ and (b) T_N_:R_H_. These groups will be subjected at an inspired fraction of O_2_ (FiO_2_) to simulate approximately 3000 m of altitude in the Multisport Gymnasium. The other training group will train in normoxia (T_N_:R_N_) at FiO_2_ corresponding to sea level in the Multisport Gymnasium. Hypoxia generators will be used together with the normobaric hypoxia tents. It is important to note that O_2_ saturation will be monitored during all training sessions, which will allow us to verify the response to hypoxia during training and the application of the Lake Louise Scale.

All sessions will be developed with a group of researchers and monitored by acute responses to the training using HR, blood oxygen saturation (SPO_2_), and RPE. Weekly, acute responses will be associated with exposure to hypoxia using a questionnaire. These data will be analyzed as soon as they are collected to provide the highest safety to the participants. Any unexpected responses will be communicated to the study’s coordinator, who will be available at all the time used for intervention and evaluations. In addition, on weeks 2, 4, and 6, the blood [La-] will be evaluated to control the training load.

### Acute responses

#### Blood oxygen saturation (SPO_2_)

Blood O_2_ saturation will be monitored using a pulse oximeter (G-Tech Portable). The equipment will be positioned on the participants’ distal phalanx during training, and the measurements will be recorded in four moments, id. est., rest, end of each stimulus, lowest result during the break, and end of the pause.

#### Rate of perceived exertion (RPE)

The training intensity will be controlled by RPE [38].

#### Acute responses associated with exposure to hypoxia

Both training groups (normoxia and hypoxia) will answer this questionnaire to maintain the impartiality of the study. The Lake Louise Score [39] will be used. There is no intention to diagnose acute mountain disease but verify and monitor the acute responses to hypoxia exposure.

#### Blood lactate concentration [La^-^]

Blood [La-] will be determined using the YSI 2300 STAT analyzer (Yellow Springs, OH, USA). For this, 25 μL of whole blood will be collected from the earlobe, using previously calibrated heparinized capillaries. Blood samples will be immediately homogenized in microtubes containing 1% sodium fluoride.

### Outcomes

The primary outcome is the maximum oxygen consumption (VO_2_peak). The secondary outcomes include lung function, inflammatory mediators, hematological, autonomic parameters, AT, body composition analysis, quality of life, mental health, anthropometric measurements, and physical fitness. All outcomes will be assessed at baseline, immediately post-intervention, and later, 4 weeks after the end of the intervention. After 4 weeks, the primary and some secondary outcomes will be assessed (partial evaluation). The primary and secondary outcomes will be presented by mean and standard deviation.

### Participant timeline

This study is ongoing and started in September 2020, ending in December 2021. The schedule for enrolment, interventions, and assessments is presented in Table 2.

**Table 2:** Schedule of enrolment, interventions, and assessments

Timepoint	Study period					
	Enrolment	Allocation	Post-allocation			
	***-t***_***1***_	0	***Baseline***	***Partial evaluation*** ***(week 5)***	***Post-training*** ***(week 9)***	***After 4 weeks of training end (week 13)***
**Enrolment**						
**Eligibility screen**	X					
**Informed consent**	X					
**Allocation**		X				
**Interventions (training)**						
***Normoxia (N)***			X			
***Hypoxia (H)***			X			
***Hypoxia recovery (HR)***			X			
***Control group***			X			
**Assessments**						
***Lung function (spirometry)***			X	X	X	X
***Autonomic parameters (HRV)***			X	X	X	X
***Max Aerobic power (VO***_***2***_***peak)***			X	X	X	X
***Anaerobic threshold***			X	X	X	X
***Inflammatory mediators***^***1***^			X		X	X
***Haematological parameters***^***2***^			X		X	X
***Body composition (iDXA)***			X		X	X
***Quality of life (SF – 12)***			X		X	X
***Mental Health (DASS–21)***			X		X	X
***Anthropometric (BMI, WC)***			X		X	X
***Physical fitness (motor tests)***			X		X	X

*HRV* heart rate variability, *max* maximum, *BMI* body mass index, *WC* waist circumference

^1^Inflammatory mediators include quantification of cytokines and profile of eicosanoids

^2^Haematological parameters include complete blood cell count, total cholesterol, triglycerides, HDL-c, LDL-c, lactate dehydrogenase, liver enzymes (alanine aminotransferase and aspartate aminotransferase), and EPO

### Sample size

For sample size estimation for clinical trial study design, using sample size estimation as comparing parallels groups (such as three training groups: (a) T_H_:R_H_, (b) T_N_:R_H_, (c) T_N_:R_N_), and control group. A power function of 80% will be used, a level of significance of 5% and an average difference between groups of 10% will be estimated. Based on the formula described previously [40], the sample size required per group is 20. Hence, the total sample size required is 80 for the four groups, considering a drop-out rate of 10%, so the total sample size required is 88, that is, 22 individuals in each group [41].

### Recruitment

The invitation will be made to the Health Institutions to offer the possibility of participating in this research to patients who have had moderate to severe symptoms approximately 30 days after the recovery of clinical signs or medical discharge (hospitalized people). Furthermore, the disclosure will happen for the entire population of Ribeirão Preto, SP, Brazil, and the region through the local television channels, USP’s local radio and news from the internet, social networks, and among other media outlets. Participants will be asked to complete the evaluations on week 0. After that, they will be assigned randomly to each group. This study will develop every effort to control confounding variables. Randomization and allocation will be performed by the research coordinator. Considering special randomization techniques, in this project, we will be using the randomization of matched pairs [42], considering a baseline pairing strategy regarding confounding variables such as sex, age, and level of physical conditioning of the participants for their selection and random assignment in the research groups. As there are three training groups, (a) T_H_:R_H_, (b) T_N_:R_H_, and (c) T_N_:R_N_, pairing groups will be organized consisting of participants based on the matching characteristics above, which will be drawn randomly using Microsoft Excel for allocation to groups. This method is satisfactory and suitable for intervention studies in which there are not many participants. In terms of blinding, only the research coordinator is aware of the allocation. Different researchers will work on the intervention and collect information from the participants as evaluations, who will be blinded to avoid potential bias. To maintain the overall quality and legitimacy of the clinical trial, unblinding should occur only in exceptional circumstances when knowledge of the actual treatment is essential for further management of the patient.

### Data collection

#### PAR-Q and anamnesis

Firstly, following what is put for people aged between 15 and 69 in the State of São Paulo Law No. 16724 of 22 May 2018, participants will answer the Physical Activity Readiness Questionnaire (PAR-Q) and those who respond positively to any of the questions, they will sign the Term of Responsibility.

Sociodemographic data, general, and specific (COVID-19) health status related to lifestyle, information on comorbidity, and drug treatment will be assessed through the Anamnesis. Answers about the symptoms associated with COVID-19 will allow assessing the intensity of the disease’s manifestation.

### Longitudinal responses

#### Questionnaires

The International Physical Activity Questionnaire (IPAQ) - short version, validated in Brazil [43], will measure the usual level of physical activity and the Food Consumption Markers Form of the Ministry of Health [44] to assess food consumption. Participants will be instructed to maintain similar physical activity and eating habits throughout the study. The objective is to certify that the possible differences between the groups after the intervention in the variables of interest are related to the physical training intervention’s effect, not to other environmental factors.

The 12-Item Health Survey (SF-12) will assess the perception of the quality of life [45]. In addition, mental health status will be assessed using the Depression, Anxiety and Stress Scale (DASS - 21) [46], and the calculation of the score will be based on a previous study [47].

#### Autonomic parameters

HR variability (HRV) will be analyzed using the RR intervals (RR) from the Polar Team2 HR transmitter (Polar Electro Oy, Kempele, Finland) and recorded at a sampling frequency of 1000Hz. The data will be recorded and stored for further analysis. The HRV analyses will be performed using custom computer software (CardioSeries v2.0, http://sites.google.com/site/cardioseries) developed by Dias, DPM of the University of São Paulo, Brazil. The interpolated RR series will be divided into half-overlapping sets of 256 data points, overlapping 50% (Welch Protocol). The stationary segment will be visually inspected, and those with artifacts or transients will be excluded. Each RR static segment will be submitted to the Fast Fourier Transform (FFT) spectral analysis after the Hanning window [48]. The analysis of HRV will be performed by linear methods and analyzed in the domains of time (SDNN and RMSSD) and frequency (LF, HF, and LF/HF ratio) [49].

#### Anthropometric measurements

The body mass index (BMI) will be used to measure nutritional status recommended by WHO [50], and waist circumference (WC) [51] will also be measured.

#### Body composition

The dual-energy X-ray absorptiometry technique (iDXA - GE Lunar - DPX-NT) will be used to analyze body composition and the distribution of the lean, bone, and adipose tissue. The radiation dose that the participants will receive will be less than 0.05 [52], equivalent to 50 times less than an X-ray exam. When positioned on the device, the subjects will remain immobile in the supine position during the exam (about 15 min). The method will estimate body composition by dividing the body into three anatomical compartments: fat-free mass, fat mass, and bone mineral content.

#### Lung function

For pulmonary function evaluation, a portable spirometer (Micro Medical, Rochester, UK) will be used, following the current standards [53] and guidelines for pulmonary function tests [54]. Participants will be instructed to perform the manoeuvers to assess forced vital capacity (FVC) and forced expiratory volume (FEV).

#### Genotyping

Blood collection will be performed by peripheral venous access after an 8-h fast, and the DNA extraction process adopted will be Salting Out. The ECA I/D genetic variants (rs1799752) will be amplified by polymerase chain reaction (PCR) (Applied Biosystems StepOnePlus), according to a previous study [55]. In addition, the genetic variants of AGTR1 A1166C (rs5186), T>G in the EPO gene promoter region (rs1617640), and G6002A at the EPO receptor (rs121918116) will be determined by real-time polymerase chain reaction (qPCR). The reaction will be performed using an allelic discrimination assay TaqMan (CAH5I790 Thermo Fisher, USA) and TaqMan genotyping master mix (Applied Biosystems, USA).

#### Hematological parameters

Complete blood cell count, such as total erythrocyte count, hematocrit, hemoglobin concentration, mean corpuscular volume, mean corpuscular hemoglobin, mean corpuscular hemoglobin concentration, total leukocyte, platelet, and reticulocyte count will be evaluated. In addition, total cholesterol, triglycerides, HDL-c, LDL-c, lactate dehydrogenase, and liver enzymes (alanine aminotransferase and aspartate aminotransferase) will also be analyzed. The collection will be carried out at the School of Physical Education and Sport of Ribeirão Preto by a trained and specialized professional. Later, the samples will be sent for analysis at the Clinical Analysis Laboratory of the Faculty of Pharmaceutical Sciences of Ribeirão Preto, according to the technical service’s standard routine and methodology. Aiming to determine the EPO concentration in the plasma samples, the EPO Immunoassay ELISA Kit (R&D Systems, Billings, USA) will be used.

#### Dosage of inflammatory soluble protein mediators

The quantification of cytokines IL-6, IL-8, IL-10, and TNF-α will be performed by the ELISA - multiplex method according to the manufacturer’s specifications (R&D Systems, Billings, USA).

#### Inflammatory lipid mediators

The profile of eicosanoids will be evaluated in plasma by mass spectrometry (LC-MS/MS) in samples collected with EDTA anticoagulant at the facility available from the Faculty of Pharmaceutical Sciences of Ribeirão Preto - USP. The standard protocol requires the use of lipid solid-phase extraction and high-resolution multiple-reaction monitoring to develop a target bioanalytical method for eicosanoid quantification. Data acquisition and processing will be performed using PeakView^TM^ and MultiQuant^TM^ software (Sciex, Foster, CA, USA) (Sorgi CA, et al., Scientific Data, 2018).

#### Blood pressure

Blood pressure will be assessed using an automatic digital arm pressure meter (OMRON, model HEM-7113), following the “VII Brazilian Guidelines on Hypertension” [56].

### Physical fitness

#### Maximum aerobic power (VO_2_peak), anaerobic threshold (AT), and exercise intensity corresponding to VO_2_peak (iVO_2_peak)

An incremental test will be used to estimate AT, VO_2_peak, and iVO_2_peak. The warm-up will be 5 min in the mechanical cycle ergometer label *Monark* (Monark, Brazil) without load (0 Kp). At each 2-min stage, there will be an increase of 0.25 Kp (approximately 13 W) until voluntary exhaustion [27]. O_2_ consumption will be measured with each breath using the gas analyzer (K4b^2^, COSMED, Italy), calibrated according to the manufacturer’s specifications. Blood samples (25 μL) will be collected from the earlobe at each stage’s end to analyze the blood [La-]. Concomitantly, HR and RPE will be monitored at the end of each stage. VO_2_peak will be defined as the highest mean VO_2_ in the last 60 s in the test, considering at least three of the criteria: volitional exhaustion, blood [La-] ≥ 8.0 mmol, > 90% of the maximum HR predicted for age (HRmax = 220 - age), RPE ≥ 9, respiratory quotient ≥ 1.10, and inability to maintain a frequency of at least 60 rpm. The iVO_2_peak will be the lowest intensity at which the individual reaches VO_2_peak during the test. If the individual does not sustain the intensity until the stage’s end, the iVO_2_peak will be assumed like peak power (PP), estimated by the equation [57]:

$$ PP= Last\ full\ intensity+\left(\frac{Dwell\ time\  at\  last\ intensity}{Time\ of\ each\ stage}\right)\ast Increment $$

The points obtained between the blood [La-] and intensities will be subjected to two linear adjustments so that the intersection will be assumed as the intensity of AT [58].

A partial evaluation containing the incremental test will be carried out at week 5 (half of the intervention) to adjust the training load (evaluation 2). Therefore, the incremental test will be performed in four moments: evaluation 1 (week 0 - pre), evaluation 2 (week 5 - half of the intervention), evaluation 3 (week 9 - post), and evaluation 4 (week 13 - four after the intervention’s end).

#### Strength resistance of lower limbs and agility and dynamic balance

Three motor tests will be performed in the following order to assess: (i) strengh resistance of lower limbs [59], (ii) agility and dynamic balance [60], and a (iii) 6-min walk [59].

### Statistical analysis

After double data entry, an exploratory analysis of the data will be carried out. The primary objective will be to summarize the values, organize, and describe the data through tables with descriptive measures and graphs. Continuous variables will be expressed in terms of basic descriptive statistics (mean, median, and standard deviation), whereas categorical variables will be described in terms of frequency and percentage. The effects of the different types of training (hypoxia and normoxia), before, after 8 weeks, and after 4 weeks after the intervention, for continuous outcomes will be compared using a linear regression model with mixed effects (fixed and random). If there are binary outcomes, a cox proportional hazards model will be used to estimate the incidence rates and their respective confidence intervals, including potential confounders as covariates. The possible associations between hematological, immunological, autonomic, aerobic, and anaerobic variables will be verified with hypothesis testing and the use of other regression models. In the case of handling missing data, we will use statistical methods as multiple imputation or Bayesian methods. The level of significance will be set at 5% in all analyses.

### Ethics

This study was approved by the School of Physical Education and Sport of Ribeirão Preto – University of São Paulo (USP) and Faculty of Pharmaceutical Sciences of Ribeirão Preto *–* USP Research Ethics Committees (CAAE: 33783620.6.0000.5659, and CAAE: 33783620.6.3001.5403, respectively). Activities will start after signing the free and informed consent term presented by the coordinator of the study. Following the Research Ethics Committee orientations, a biorepository at the School of Physical Education and Sport of Ribeirão Preto *–* USP was created to store biological samples for use in future studies depending on the participants consent (CAAE: 33783620.6.0000.5659).

All care for the safety of the participants and the work team will be taken. First, an evaluation of the health status will be carried out. The participants who do not have limitations or discomfort that may prevent the evaluations’ performance or the intervention will participate in the proposed intervention. Data will be added to a confidential dataset, and an alphanumeric code will be assigned to each participant. All local databases will be secured with password-protected access systems.

Any modifications to the protocol which may impact on the study conduction, the potential benefit of the participant or safety, including changes of study objectives, design, procedures, participant population, sample sizes, or significant administrative aspects will require a formal amendment to the protocol and will be communicated to the Brazilian Clinical Trials Registry (RBR-5d7hkv) and the Research Ethics Committees.

Authors will create scientific reports to be submitted to peer-reviewed journals. Also, information about the different outcomes will be shared in different scientific events.

## Discussion

It is plausible that the convalescent people of COVID-19 who showed moderate to severe symptoms are in the process of gradual recovery and still have changes in their health status, even after approximately 30 days of recovery from clinical signs or medical discharge. Therefore, the monitoring control group that will not train will describe the effects of COVID-19 on lung function, hematological, immunological, autonomic parameters, physical fitness, and mental health.

The gradual recovery process of the participants will be observed according to the association of training (T) and recovery (R) with hypoxia (H) or normoxia (N). Therefore, it is expected that the normoxia training and recovery group (T_N_:R_N_) can show better results compared to the control group. Besides, both groups that will train in hypoxia may demonstrate even better results than the T_N_:R_N_ group, with a higher result for the group that will perform the training in normoxia and the recovery in hypoxia (T_N_:R_H_), comparing the group that will perform training and recovery in hypoxia (T_H_:R_H_). The justification for these two groups to train differently associated with hypoxia is that exercise intensity can be reduced with hypoxia exposure. In this way, to minimize this possibility, the group that will breathe hypoxia air only during the pause could perform the training stimulus in greater intensity (normoxia).

The expectation is that physical activity can be used to face the COVID-19 pandemic and other health problems as a complementary treatment. The consolidated evidence on the benefits of physical activity for physical, physiological, social, and mental health and the knowledge generated by this research project may contribute to solving priority health problems in Brazil. Furthermore, this project’s results may contribute to the qualification of health care practices and promote scientific, technological, and innovation development in the Health area, aiming at strengthening the Unified Health System of Brazil.

Still, it is expected that the genetic variants studied may be associated with the recovery process of the convalescents of COVID-19 and with the effect of moderate training combined with intermittent normobaric hypoxia. The study of genetic variants can bring important advances in understanding the variation in responses related to the triggering and progression of some diseases and the effect of physical training.

To improve retention, we will provide (i) written feedback to all participants about the assessments results at the beginning, after 4, 8, and 12 weeks of the intervention; (ii) periodic communications and presentations to inform the current status of the study, plans for the next phase, and motivating the participation; and (iii) flexible as possible with training sessions and evaluations schedule.

Accessing participants can be difficult due to the social isolation imposed by the pandemic. However, this limitation can be minimized by emphasizing the benefits that the intervention proposed by the present study can promote and emphasizing that all safety-related care will be taken.

The pandemic caused by the SARS-CoV2 virus is not yet under control, and the state of global emergency in Public Health remains declared. The number of deaths and new cases remains high. Even though the recovery rate is high, changes are observed in recovered patients even after 30 days of the interruption of clinical signs or medical discharge, in addition to some undeclared sequelae. Thus, a moderate-intensity interval training program performed in intermittent normobaric hypoxia could be an efficient alternative in rehabilitating convalescents who presented moderate to severe symptoms to recover lung function, physical fitness, quality of life, and mental health, as well as immunological homeostasis, preventing chronic and autoimmune inflammatory diseases, and preventing new infections. And the study of genetic variants can provide important advances in a better understanding of disease progression variability and proposed treatments.

### Strengths and limitations

Some points strengthen and support this proposal:

- Consolidated evidence on the benefits of physical training for health promotion, prevention, and control of various diseases
- Decreased number and reduction in the severity of COVID-19 cases in cities with higher altitude
- Use an innovative hypoxia utilization protocol as an additional training stimulus, applicable to the participants’ real-life context.
- Increased EPO’s production, stimulated by HIF-1α, recent scientific relevance in Physiology and Medicine, with benefits that can counter the harmful effects of COVID-19

Regarding limitations, it may be challenging to access participants due to social isolation in the pandemic context.

### Trial status

Recruiting started in September 2020 and is expected to be completed in December 2021. The current protocol is version 4, approved by the ethical committee on 14 August 2020. Currently (16 June 2020), we included 84 participants.

## Supplementary Information

**Additional file 1.**

## Data Availability

The datasets used and/or analyzed during the current study will be made available from the corresponding author upon reasonable request.

## Abbreviations

ACE: Angiotensin-converting enzyme
AGTR1: Angiotensin II type 1 receptor
AT: Anaerobic threshold
AU: Arbitrary units
BMI: Body mass index
DASS–21: Depression, Anxiety and Stress Scale
EPO: Erythropoietin
FEV: Forced expiratory volume
FiO_2_: Inspired fraction of O_2_
FVC: Forced vital capacity
H: Hypoxia
HIF-1α: Hypoxia-inducible-factor-1α
HRV: Heart rate variability
IPAQ: International Physical Activity Questionnaire
iVO_2_peak: Exercise intensity corresponding to VO_2_peak
[La-]: Blood lactate concentration
N: Normoxia
O_2_: Oxygen
PAR-Q: Physical Activity Readiness Questionnaire
PCR: Polymerase chain reaction
PP: Peak power
qPCR: Real-time polymerase chain reaction
R: Recovery
RPE: The rate of perceived exertion
SF-12: 12-Item Health Survey
SPO_2_: Blood oxygen saturation
T: Training
Trimp: Internal training load
USP: University of Sao Paulo
VO_2_peak: Maximum oxygen consumption
WC: Waist circumference
