# Supplementary material for: Effects of moderate-intensity intermittent hypoxic training on health outcomes of patients recovered from COVID-19: the AEROBICOVID study protocol for a randomized controlled trial
Source: Trials. 2021 Aug 12;22:534. doi: 10.1186/s13063-021-05414-2 (PMC8358903; doi:10.1186/s13063-021-05414-2)
Supplement: Supplementary file 1 — Additional file 1. [file 13063_2021_5414_MOESM1_ESM.docx]

**APPENDIX**

**Item 2b** – Table: Trial registration data

| **Data category** | **Information** |
| --- | --- |
| Primary registry and trial identifying number | Brazilian Clinical Trials Registry - RBR-5d7hkv |
| Secondary identifying numbers | UTN code: U1111-1260-7479  CAAE: 33783620.6.0000.5659  CAAE: 33783620.6.3001.5403 |
| Date of registration in primary registry | 03 November 2020 |
| Source(s) of monetary or material support | School of Physical Education and Sport of Ribeirão Preto |
| Primary sponsor | School of Physical Education and Sport of Ribeirão Preto |
| Secondary sponsor(s) | USP Vida Project (3518 -2020) |
| Contact for public queries | PhD Atila Alexandre Trapé – atrape@usp.br |
| Contact for scientific queries | PhD Atila Alexandre Trapé – atrape@usp.br |
| Public title | Effect of Moderate Physical Training on a bicycle associated with altitude simulation on the health status of people recovered from covid-19 (AEROBICOVID) |
| Scientific title | Effect of moderate-intensity training associated with normobaric hypoxia on lung function, haematological, immunological, autonomic parameters and related to physical fitness in convalescent people from covid-19 (AEROBICOVID) |
| Countries of recruitment | Brazil |
| Health condition(s) or problem(s) studied | Coronavirus infections, respiratory function, immunologic factors, cardiorespiratory fitness, physical fitness, hypoxia-inducible factor 1, alpha subunit |
| Intervention(s) | The physical training groups will be randomly divided according to the association of training (T) and recovery ® with hypoxia (H) or normoxia (N): a) TH + RH, b) TN + RH, c) TN + RN |
| Key inclusion and exclusion criteria | The inclusion criteria will be (1) participants aged between 30 and 69 years old and convalescent from covid-19 (having the test with a positive diagnosis), (2) having moderate to severe symptoms, (3) approximately 30 days since recovery from clinical signs or medical discharge (if they had been hospitalised).  The exclusion criteria will be (1) exposure to high-altitude places > 1,500 m in the last three months, (2) significant physical limitations to carry out assessments or intervention, (3) acute or chronic clinical illnesses without medical supervision, (4) anaemia, (5) use of immunosuppressive drugs, (6) pregnant women, (7) hormone replacement, (8) smokers, and (9) excessive use of alcohol or drugs. |
| Study type | A clinical trial of treatment, prospective, randomized, controlled, parallel, double-blind, |
| Date of first enrolment | 16 September 2020 |
| Target sample size | 80 |
| Recruitment status | Recruiting |
| Primary outcome(s) | Lung function, inflammatory mediators, haematological, autonomic parameters, maximum oxygen consumption (VO_2_peak), AT, and body composition analysis. |
| Key secondary outcomes | Quality of life, mental health, anthropometric measurements, and physical fitness. |

**Item 5b**

- Trial Sponsor: School of Physical Education and Sport of Ribeirão Preto, University of Sao Paulo
- Contact name: PhD Cristiano Roque Antunes Barreira (Director)
- Address: Avenida Bandeirantes, 3900 – Monte Alegre – Ribeirão Preto/SP - CEP 14040 – 907
- Telephone: +551655150529
- Email: eeferp@usp.br

Item 5c – “The sponsor and the funder (USP Vida Project) had no role in the design of this study and will not have any role during its execution, analyses, interpretation of the data, or decision to submit results.

Item 5d –

*Principal investigator*

- Design and conduct the study
- Preparation of protocol and revisions
- Organising steering committee meetings
- Publication of study reports
- Members of Trial Management Committee (TMC)
- Steering committee (SC)
- Agreement of final protocol
- Recruitment of patients
- Reviewing progress of study and if necessary, agreeing changes to the protocol

*Trial management committee (TMC)*

- Study planning
- Organisation of Trial Steering Committee (TSC) meetings
- Budget administration
- Advice for lead investigators
- Data verification
- Randomisation
- Data manager
- Data verification

*Lead investigators*

- Identification, recruitment, and data collection of the study
- Follow up of study patients and adherence to study protocol
- TSC members

21a – Monitoring Committee

A Data Monitoring Committee (DMC) will be established. The DMC will be independent of the study organisers. During the period of recruitment to the study, interim analyses will be supplied, in strict confidence, to the DMC, together with any other analyses that the committee may request. This may include analyses of data from other comparable trials. In the light of these interim analyses, the DMC will advise the TSC.

21b - An interim analysis will be performed on the primary endpoint when 40 participants have been randomised and have completed the eight weeks of physical training. The interim analysis will be performed by an independent statistician, blinded for the treatment allocation (different groups of training). The statistician will report to the independent data and safety monitoring committee (DSMC). The DSMC will have unblinded access to all data and will discuss the results of the interim analysis with the TSC in a joint meeting. The TSC decides on the continuation of the trial and will report to the central ethics committee.

23 – Auditing

Data monitoring and quality assurance through the combination of our web-based, instantaneous electronic validation, the Data Coordinating Centre (DCC) daily visual cross-validation of the data for complex errors, and regular on-site monitoring, the quality and completeness of the data will be reflective of the state of the art in clinical trials. The primary objectives of the DCC during the on-site visits are to educate, support and solve problems. The monitors will discuss the protocol in detail and identify and clarify any areas of weakness. At the start of the trial, the monitors will conduct a tutorial on the web-based data entry system. The coordinators will practice entering data so that the monitors can confirm that the coordinators are proficient in all aspects of data entry, query response, and communication with the DCC. They will audit the overall quality and completeness of the data, examine source documents, interview investigators and coordinators, and confirm that the clinical centre has complied with the requirements of the protocol. The monitors will verify that all adverse events were documented in the correct format and are consistent with protocol definition. The monitors will review the source documents as needed, to determine whether the data reported in the Web-based system are complete and accurate.

The focus of the visit/electronic monitoring will be on source document review and confirmation of adverse events. The monitor will verify the following variables for all patients: initials, date of birth, sex, signed informed consent, eligibility criteria, date of randomisation, treatment assignment, adverse events, and endpoints.

Item 29 - Intra-Study Data Sharing

The Data Management Coordinating Centre will oversee the intra-study data sharing process, with input from the Data Management Subcommittee. All Principal Investigators will be given access to the cleaned data sets. Project data sets will be housed on the Project Accept Web site and/or the file transfer protocol site created for the study, and all data sets will be password protected. Project Principal Investigators will have direct access to their own site’s data sets and will have access to other sites data by request. To ensure confidentiality, data dispersed to project team members will be blinded of any identifying participant information.”

Item 30 - The work team is trained and qualified to attend first aid and the University of São Paulo offers transportation to a nearest Emergency Health Unit in case of need for specialized assistance during physical training assessments and intervention.

Item 31b - The publications subcommittee will review all publications following the guidelines given below and report its recommendations to the Steering Committee.

- Data analysis and release of results

- Review process

- Primary outcome papers

- Other study papers, abstracts, and presentations

Item 31c - Data sharing statement No later than 3 years after the collection of the 1-year post randomisation interviews, we will deliver a completely deidentified data set to an appropriate data archive for sharing purposes.

Item 32 – Free and clarified consent

We invite you to participate in this study under the responsibility of a group of researchers and coordinated by PhD Átila Alexandre Trapé, entitled “Effect of moderate-intensity training associated with normobaric hypoxia on lung function, haematological, immunological, autonomic parameters, and related to physical fitness in convalescent people from covid-19 (AEROBICOVID)”, to be held at the Laboratory of the Olympic Gymnasium of the Physical Education School of Ribeirão Preto (EEFERP), part of the University of São Paulo (USP).

First, we would like to clarify some terms related to our project. Other clarifications will appear during this document always after this arrow (
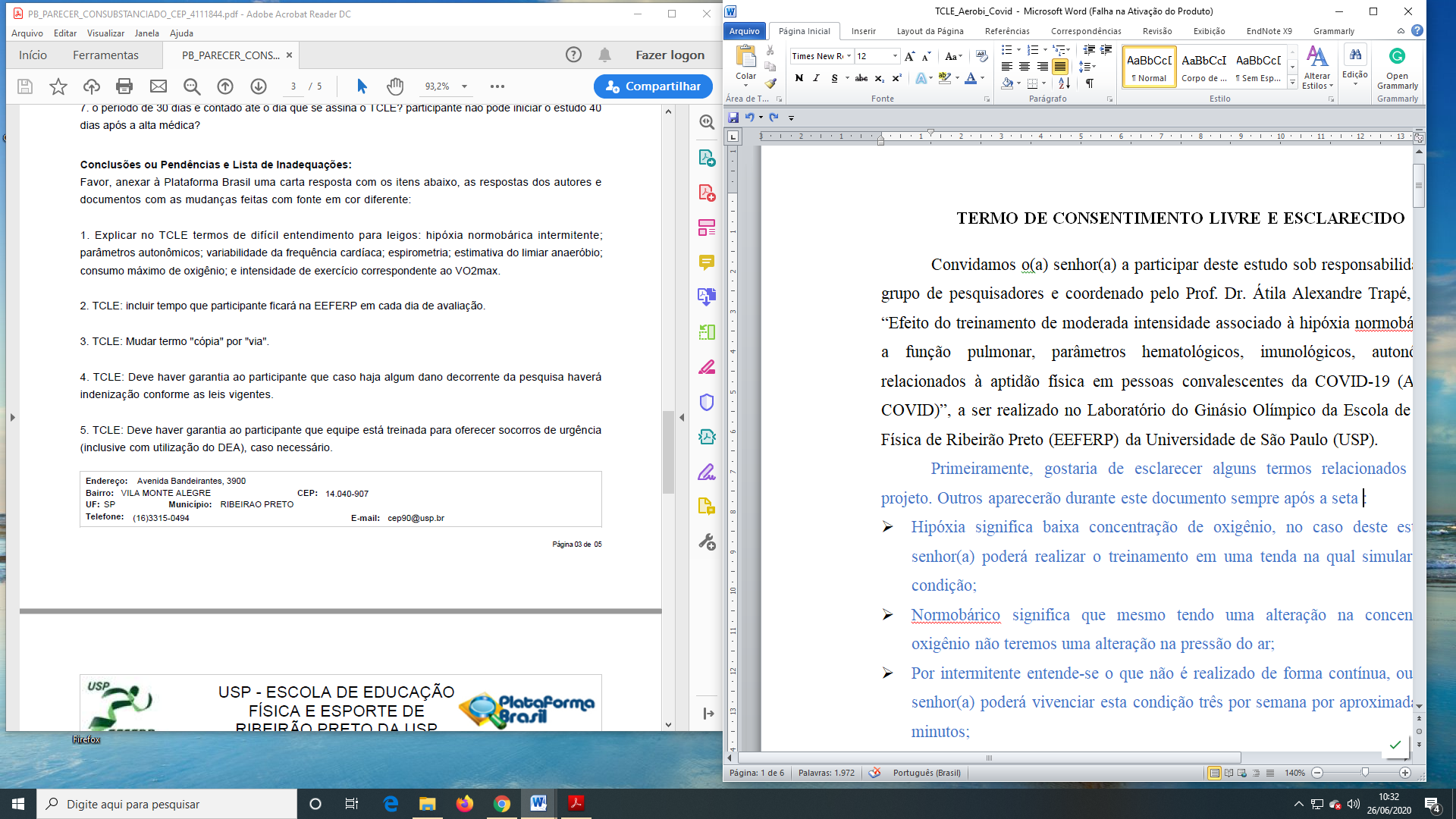
), as the words appear:

- Hypoxia means low oxygen concentration. In the case of this study, you will be able to carry out training in a tent, where we will simulate this condition as if you were in a higher city, around 2000 - 3000 meters (6561.68 – 9842.52 ft) - above sea ​​level;
- Normobaric means that even if there is a change in the oxygen concentration, there will be no change in air pressure;
- The autonomic parameters are related to the Nervous System and the influence that this system can have on cardiovascular aspects. This measurement will be performed through the heart rate variability, which can be understood as the measurement of time between two consecutive heartbeats;

This document provides information about the project, as well as information about potential risks and benefits. In it, your rights as a participant in an experimental study are detailed. Please read this document carefully, and ask about any questions related to your participation in the study.

The study has two main objectives: 1. To describe the health status of people convalescent from covid-19 who presented moderate symptoms through lung function, haematological (blood), immunological and autonomic parameters, and related to physical fitness and mental health; and, 2. To analyse the effects of moderate-intensity interval training on intermittent normobaric hypoxia on lung function, haematological, immunological, autonomic parameters, and those related to physical fitness and mental health in convalescent people from covid-19 who presented moderate symptoms.

Regarding the symptoms, you will answer a questionnaire that will ask about various symptoms during the infection by covid-19 in your body. Severe symptoms led to significant limitations and, therefore, hospitalisation and use of respirators. Mild symptoms were like a common cold, without interfering much in your mood. In this study, our idea is to work with people who have had moderate symptoms, something intermediate between the mild and severe ones explained earlier. Thus, two situations can be included in the moderate symptoms: those who were followed up at home and lost their mood, disposition, had a fever and moderately experienced other symptoms; and also those who needed to be hospitalised but did not need a respirator.

- Intermittent means what is not performed continuously, that is, you can experience this condition three times a week for approximately 50 minutes;

This study is essential to understand the disease and to investigate whether moderate-intensity physical training performed under hypoxia or in normoxia (the normal oxygen concentration, as found in a city close to sea level) can be a tool for a gradual recovery in people recovered from covid-19. Advances in the health area occur through research like this and, hence, your participation is important.

To participate in this research, you must: be over 18 years of age, having tested positive for covid-19, showing moderate to severe symptoms in the manifestation of the disease, and present 30 days after recovery of clinical signs related to covid-19 or medical discharge. Initially, you will be subjected to a health screening to analyse if you do not have the health conditions that indicate the performance of one or more tests and if you can perform the training intervention. Some health conditions contraindicate the participation of one or more of the tests, such as problems in the muscles and bones, presence of a symptom that suggests cardiovascular disease without medical supervision, and presence of cardiovascular disease or risk factors for such diseases without having been tested the maximum ergometric with an electrocardiogram (the one in which we run or pedal to the highest speed we can with electrodes on the trunk to analyse the electrical activity of the heart). And, if any of the evaluation results that may indicate a health problem other than that caused by covid-19, our team will contact you to deliver the exam result, and to guide you a follow-up at a Health Unity in the city of Ribeirão Preto/SP.

Regarding the evaluations, you will remain at EEFERP-USP for approximately 1 hour each day. On the first day, you will answer the Anamnesis with questions about personal data, general and specific health status about covid-19, as well as lifestyle, comorbidity information (diseases), and drug treatment. It will also answer questionnaires on the practice of physical activity, eating habits and mental health; afterwards, the anthropometric assessment (body mass, height, and waist), blood pressure measurement, in addition to a first part of the information and practice for the third-day evaluation. On the second day, after eight hours of fasting, you will perform blood collection (approximately 16 ml) for analysis of haematological parameters and iDXA for estimating body composition (lying on a kind of stretcher while a device will move slowly above your body as if it were scanning). This equipment makes use of a non-lethal dose of radiation (50 times smaller than a hospital x-ray). Still, on the second day, you will perform a second part of the familiarisation of the third-day evaluation. On the third day, resistance test of muscular strength of lower limbs, the test of agility and dynamic balance, spirometry to assess lung function, and the graded test with increased load on the bicycle to estimate the anaerobic threshold (AT), maximum oxygen consumption (VO_2_peak) and exercise intensity corresponding to VO_2_peak (iVO_2_peak) in Watts (W max). All of these evaluations will take place at the Olympic Gymnasium of EEFERP - USP.

- Spirometry, or pulmonary function test, is an evaluation performed using a mouthpiece connected to a machine (spirometer). Between the steps, we will ask you to breathe calmly, fill your chest and blow with maximum strength;
- The estimate of the anaerobic threshold is related to the measurement of lactate, which is a substance that our body produces in response to metabolism, and can change at different exercise intensities;
- Maximum oxygen consumption (VO_2_peak) represents aerobic capacity, related to cardiorespiratory capacity;
- Based on the VO_2_peak result, you will perform the training at a moderate intensity of exercise corresponding to what was found in this assessment. The 5 min stimulus in each series will be in what we call training zone 2, at 90 to 100% of the anaerobic threshold. And we will have 2.5 min breaks, that is, the stimulus and pause ratio is 2: 1.

During the incremental test, and also during training, we will analyse HR variability, blood oxygen saturation, rate of perceived effort (RPE), and blood lactate concentrations, before, during and immediately after the sessions.

The HR variability assessment will be made using a tape placed around your chest that will capture your heart rate, the analysis will take place for 10 minutes, and you will be lying supine (belly up) on a stretcher. Oxygen saturation and heart rate (during the training session) will be measured using a small device (portable oximeter) that will be attached to the tip of your index finger for a few seconds. The RPE is an instrument by which you will indicate the sensation of the effort made on a scale from 0 to 10. Blood lactate concentrations will serve to obtain its anaerobic threshold. For blood lactate analysis, after abrasive cleaning with cotton and alcohol, your ear will receive a small needle prick, and a drop of blood will be punctured into a capillary that will later be analysed.

After carrying out the initial evaluation, explained in the previous paragraph, if you are interested in, you will start the program of moderate-intensity physical training on bicycles, which will last for eight weeks, three sessions per week, 45 minutes each session. This intervention will be held individually at EEFERP - USP, and all evaluations will be repeated 9 and 13 weeks after the beginning of your participation. You will be able to train in hypoxia or normoxia; both groups will perform the same training in the same tent. Only the concentration of available oxygen inside the tent will change. As this is a “blind” study, participants will not know if they are training in hypoxia or normoxia. Also, if you are available to do the evaluations but not to perform the intervention, you will be invited to be part of the control group, and have a follow-up of your health.

It is widely known that regular physical exercise has a positive impact on the health and quality of life of any individual. Therefore, the orientation after participating in this project is for you to continue with an active lifestyle. We will indicate possibilities in extension projects at USP, by Ribeirão Preto’s city government, among others. Another benefit of this study will be the generation of scientific knowledge. Also, you will receive a summary of the evaluations carried out in a printed document.

The general risks of this study are small and are associated with evaluations and training intervention (disturbances or discomfort caused by pain during and after exercise or exposure to hypoxia). The experimental situations will be performed in environments with strictly controlled conditions and compatible with the prescribed effort, already used in previous research. During all the practical activities, there will be the supervision of a trained and enabled team to provide emergency first aid. Blood collection will be performed at EEFERP-USP by a qualified and experienced professional, using sterile and disposable material. The risks of this collection will be minimal, with a small chance of you feeling pain, of ecchymosis or simple swelling on your arm at the collection site, which should disappear in a few days. The collected samples will be sent later for analysis at the Faculty of Pharmaceutical Sciences of Ribeirão Preto - USP, but you will not need to go there. A bank to store samples will be created, and future analyses for other purposes may be made, depending on a supplement to the project and approval by the Ethics and Research Committee. Thus, by accepting to participate in this research project, you authorise the collection, deposit, storage and use in this current and future researches, with no further consent to the latter.

Your withdrawal from the study is guaranteed at any stage, including the storage of biological material in a biobank or biorepository, and it will be up to the responsible researcher, at any time, to proceed with its exclusion if necessary for your well-being.

Your personal information obtained in this study will be analysed in conjunction with that obtained with other patients, thus safeguarding the confidentiality of your participation. You will be updated about partial and final research results.

It is also relevant to clarify that the only expense that may happen with your participation is related to your transportation to EEFERP-USP, which will not be reimbursed. There is no financial compensation for your participation; besides, the researcher is committed to using the data only for research.

In case of any complications during the procedures, we have an automatic defibrillator model HeartStart FRx (Philips®), and we have an agreement with Medicar. Also, all processes will be followed by professionals with training in basic life support. The team is trained to provide emergency aid. If damage occurs related to the research, there will be compensated according to the laws in force.

It is important to discuss with PhD Átila Alexandre Trapé about your doubts regarding the project, its objectives, the procedures to be used, and the guarantees of confidentiality and permanent clarification before deciding to participate or not in the project. Supposing that you voluntarily agree to participate in this study, you can withdraw your consent at any time, before or during the study, without any penalties, or loss of any benefit you may have acquired.

Finally, bear in mind that this study was submitted to the Ethics and Research Committee (CEP), receiving approval on 16 July 2020. Its performance is following the Norms of the National Health Council (Resolution 466/2012), which ensure protection for volunteers involved in biomedical research.

If you have any questions or want to have access to the results of this and future studies, as well as receive guidance on their implications, please contact the researcher responsible for the study: PhD Átila Alexandre Trapé, from EEFERP-USP (Avenida dos Bandeirantes, 3900, Monte Alegre, 14040-907, Ribeirão Preto, SP, Brazil, telephone +551633158617 and +5516988305017, email: [atrape@usp.br](mailto:atrape@usp.br)).

CONSENT

I believe I have been sufficiently informed about the information that I read or was read to me describing the study. I know that I can refuse to participate in the research or that I can abandon it at any time, without any embarrassment. I received one copy of this document that was signed in two identical copies. Therefore, I give my consent to participate in the experiments of this study.

Ribeirão Preto, _____ ____________________ ________

Full name of the research participant or guardian

Signature of research participant or guardian

___________________________________________________

PhD Átila Alexandre Trapé

Responsible Researcher

If you have any questions about ethical research issues, please contact the Research Ethics Committee of the Ribeirão Preto School of Physical Education and Sport - USP: Avenida Bandeirantes, 3900 - 14040-907 - Ribeirão Preto - SP - Brazil Phone: +55163315-0494. Email: cep90@usp.br
